# Tapering Practices of Strongman Athletes: Test-Retest Reliability Study

**DOI:** 10.2196/resprot.8522

**Published:** 2017-10-31

**Authors:** Paul W Winwood, Hayden J Pritchard, Justin WL Keogh

**Affiliations:** ^1^ Department of Sport and Recreation Faculty of Community Wellbeing and Development Toi Ohomai Institute of Technology Tauranga New Zealand; ^2^ Sport Performance Research in New Zealand AUT Millennium Institute AUT University Auckland New Zealand; ^3^ Faculty of Health & Sciences Department of Exercise & Wellness Universal College of Learning Palmerston North New Zealand; ^4^ Faculty of Health Sciences and Medicine Bond University Gold Coast Australia; ^5^ Cluster for Health Improvement Faculty of Science, Health, Education and Engineering University of the Sunshine Coast Queensland Australia

**Keywords:** Internet, survey, periodization, implement training, resistance training

## Abstract

**Background:**

Little is currently known about the tapering practices of strongman athletes. We have developed an Internet-based comprehensive self-report questionnaire examining the training and tapering practices of strongman athletes.

**Objective:**

The objective of this study was to document the test-retest reliability of questions associated with the Internet-based comprehensive self-report questionnaire on the tapering practices of strongman athletes. The information will provide insight on the reliability and usefulness of the online questionnaire for use with strongman athletes.

**Methods:**

Invitations to complete an Internet questionnaire were sent via Facebook Messenger to identified strongman athletes. The survey consisted of four main areas of inquiry, including demographics and background information, training practices, tapering, and tapering practices. Of the 454 athletes that completed the survey over the 8-week period, 130 athletes responded on Facebook Messenger indicating that they intended to complete, or had completed, the survey. These participants were asked if they could complete the online questionnaire a second time for a test-retest reliability analysis. Sixty-four athletes (mean age 33.3 years, standard deviation [SD] 7.7; mean height 178.2 cm, SD 11.0; mean body mass 103.7 kg, SD 24.8) accepted this invitation and completed the survey for the second time after a minimum 7-day period from the date of their first completion. Agreement between athlete responses was measured using intraclass correlation coefficients (ICCs) and kappa statistics. Confidence intervals (at 95%) were reported for all measures and significance was set at *P*<.05.

**Results:**

Test-retest reliability for demographic and training practices items were significant (*P*<.001) and showed excellent (ICC range=.84 to .98) and fair to almost perfect agreement (κ range=.37-.85). Moderate to excellent agreements (ICC range=.56-.84; *P*<.01) were observed for all tapering practice measures except for the number of days athletes started their usual taper before a strongman competition (ICC=.30). When the number of days were categorized with additional analyses, moderate reliability was observed (κ=.43; *P*<.001). Fair to substantial agreement was observed for the majority of tapering practices measures (κrange=.38-.73; *P*<.001) except for how training frequency (κ=.26) and the percentage and type of resistance training performed, which changed in the taper (κ=.20). Good to excellent agreement (ICC=.62-.93; *P*<.05) was observed for items relating to strongman events and traditional exercises performed during the taper. Only the time at which the Farmer’s Walk was last performed before competition showed poor reliability (ICC=.27).

**Conclusions:**

We have developed a low cost, self-reported, online retrospective questionnaire, which provided stable and reliable answers for most of the demographic, training, and tapering practice questions. The results of this study support the inferences drawn from the Tapering Practices of Strongman Athletes Study.

## Introduction

The sport of strongman is relatively new and is similar to the sports of weightlifting and powerlifting, where training is primarily focused on the improvement of maximal strength and power to improve competition performance [1-3]. Unlike the sports of weightlifting and powerlifting, substantial between-competition differences can be observed in the types of events, required distances for carrying events, and incorporation of one repetition maximum (1RM) events versus repetitions with a given load. Such between-competition differences would appear similar to those that are experienced by CrossFit athletes [4], which may therefore influence the way strongman athletes taper for strongman competitions. The taper is the final period of an athlete’s training before a major competition and is of paramount importance to performance and the outcome of the event [5-8]. Winwood et al [1] found that 80% of strongman athletes incorporated some form of periodization into their training, which suggests that the majority of strongman competitors design their training to emphasize particular adaptations with the goal of increasing physical performance. As strongman and weightlifting athletes may be at greater risk for injury during competition compared to training [9], a successful taper that allows strongman athletes to recover from their recent training stressors may also reduce their risk of in-competition injury. Little scientific research currently exists regarding how to taper for strength sports, and no research exists on how strongman athletes taper for strongman competitions.

In recent years Internet-based comprehensive self-report questionnaires have been administered among strongman athletes [1,10] and strength and conditioning coaches [11]. Such surveys have elicited high response rates and provided valuable information on how strongman competitors train, the injury epidemiology associated with strongman training, and how coaches utilize strongman implements in the training of their athletes. However, a limitation to these studies was that no data were reported to verify the reliability of the survey items. Reliability refers to the consistency of answers obtained by the same respondent when a measurement is repeated on different occasions [12,13]. Test-retest reliability is measured by having the same respondents complete a survey at two different points in time to see how stable their responses are [14]. Researchers have recommended the intraclass correlation coefficient (ICC) for assessing reliability of continuous data [15-17], along with the kappa statistic, which provides a measure of agreement for categorical data corrected for chance [15,18,19].

Previous studies that have tested the reliability of Internet survey methods have demonstrated that Web-based methods are reliable [14,20-22] and can be more suitable alternatives to traditional methods [21,22]. Such studies strengthen the scientific rigor of collecting information via the Internet. Internet-based surveys have the potential to reach populations of interest across the globe, are cost efficient, and have the advantage of minimizing data collection and entry errors [21]. The popular use of social media sites (eg, Facebook) and access to the Internet via smartphones and tablets have further increased survey accessibility for respondents, which could enhance response rates.

Information on the reliability of the Internet assessment method for use with strength athletes, including the strongman population, is currently lacking. *The Tapering Practices of Strongman Athletes* survey created for this study was based on nine interview questions used with powerlifters [23,24]. Our tapering practice questionnaire has included the addition of several questions, as well as changes to the wording of original questions used in previous studies [23,24]. Based on these changes, it has become desirable to conduct a reliability study of the updated questionnaire. The present study assessed the test-retest reliability of the questionnaire on a large and diverse sample group of strongman athletes. It was hypothesized that the questionnaire would be a reliable measure for assessing the training and tapering practices of strongman athletes.

## Methods

### Participant Recruitment and Inclusion Criteria

Strongmen athletes were recruited through professional networks and multimedia methods similar to previously described procedures [1,10]. The networking site Facebook was the primary method used to recruit the strongman athletes, and identified strongman athletes were sent a letter via Facebook Messenger. The letter contained an invitation to participate in the research and the link to the online survey. Presidents of strongman clubs in New Zealand, Australia, Europe, the United States, and the United Kingdom were contacted to email the survey to their club members. The survey was available in two language options (English and Russian). An information sheet outlining the objectives and purpose of the study was situated on the first page of the online survey. Participants were asked to indicate their consent by participating in the survey. The software that was used allowed participants to exit the survey at any time and complete it at a later date, allowing participants to provide their data at the time most suitable to them. Surveygizmo [25] was used to launch the electronic survey on the Internet. The methods and procedures used in this study were approved by the Toi Ohomai Institute of Technology Research Committee (R17/05).

Participant inclusion criteria included strongman athletes who were between 18 and 65 years of age and had competed in at least one strongman competition. The criterion for a completed survey was that the participants completed the first three sections of the questionnaire on demographics, training practices, and tapering.

### Research Instrument

Strongman athletes completed a self-reported 4-page retrospective *Tapering Practices of Strongman Athletes Survey* created for this study, which was based on interview questions used with powerlifters [23,24]. The original *Strongman Tapering Practices Survey* was pilot tested with university professors and strongman athletes to ensure its ease of use with this population. As a result of pilot testing, the survey was slightly modified, including clarifications and improvements to the wording of a small number of questions, before it was administered online.

The *Strongman Tapering Practices Survey* consisted of four main areas of inquiry, including demographics and background information, training practices, tapering, and tapering practices. Demographic and background information included questions on gender, age, height, body mass, resistance training experience, strongman training experience, and competitive level. The training practices section included questions pertaining to frequency, duration, and types of training. Types of training were categorized as cardiovascular training (aerobic and anaerobic), strongman implement training, and traditional training. Strongman implement training was defined as exercises using any nontraditional training implements (eg, stones, tires). Traditional exercises were standard exercises performed in the gym by regular weight trainers and strength athletes (eg, squat, bench press). Participants were requested to detail their common/typical values for each question. For the tapering section, athletes were asked to indicate if they utilized a taper or not and their reasons why. The tapering practices section included questions on taper length and type, strategies used, and how training altered during the taper (ie, volume, intensity, duration, type of training performed, and when last performed before competition). Tapering was defined as, “a reduction in training volume over a period of time prior to a strongman event or strongman events.” Classifications of tapering (ie, step taper, linear taper, and exponential taper with a slow or fast decay) were defined according to the taper types previously described and applied [26]. Closed questions were used for Sections 1 and 2, with open and closed questions used for Section 3.

### Response Rate and Reliability Data

During the 8-week period in which the survey was open, 690 participants accessed the online survey, which included those that observed the survey, partially completed the survey, and the 454 that completed the survey. The key questions from the questionnaire selected for test-retest reliability are presented in Multimedia Appendix 1.

One hundred and thirty participants responded on Facebook Messenger indicating that they were going to complete the survey, or had already completed the survey. These participants were sent an email via Facebook Messenger and asked if they could complete the online questionnaire again for a test-retest reliability analysis. Of these 130 participants, 64 strongman athletes (49.2% response rate) accepted this invitation and completed the survey for the second time after a minimum 7-day period from the date of their first completion. To distinguish this dataset from other survey responses, participants indicated their demographic data (ie, age, height, body mass, and country of birth) so their retest survey responses could be identified and matched to their initial survey response. A test-retest analysis was done on this dataset to determine the reliability of the online strongman tapering practices questionnaire. No participants responded to the Russian language option, so the reliability study was only conducted on the English language survey.

### Data Analyses

Descriptive statistics were used to describe the cohort characteristics. Test-retest reliability for dichotomous and categorical data was computed using the kappa statistic with asymptotic standard error [27]. The kappa statistic was chosen because it is more robust than percent agreement alone, as it takes agreement by chance into consideration. Reliability was then rated using the scale developed by Landis and Koch for the purposes of comparing the reliability of the questions [27]. Reliability of the kappa statistic was rated as poor (below .00), slight (.00-.20), fair (.21-.40), moderate (.41-.60), substantial (.61-.80), or almost perfect (.81-1.00). Any missing values were indicated as *excluded* in the analyses. Reliability of continuous measures was evaluated by ICCs using a two-way random effects model, absolute agreement, and average measures ICC [28]. ICCs were classified as follows: poor (<.40), moderate (.41-.60), good (.61-.80), or excellent (>.81) [29,30]. Confidence intervals (at 95%) were calculated for all reliability measures. Data were collected using SurveyGizmo [25] and analyses were conducted using SPSS 22.0 for Windows (SPSS Inc., Chicago, IL, USA). Significance levels were set at *P*<.05.

## Results

### Cohort Characteristics

Sixty-four participants completed the online survey twice over the 8-week period. Participants were between 20 and 54 years of age, with a distribution of 46 males (46/64, 72%) and 18 females (18/64, 28%). Thirty-six participants (36/64, 56%) had competed in national amateur strongman championships, 17 (17/36, 27%) had competed at the local or regional level, and 11 (11/64, 17%) athletes had competed professionally (Table 1).

### Test-Retest Reliability Results

The test-retest reliability of continuous data for demographics and training practices of all strongman athletes (N=64) is reported in Table 2. Significant correlations (*P*<.001) were observed for all measures and reliability was excellent for all questions (ICCs=.84 to .98).

**Table 1:** Cohort characteristics

Characteristics	All participants, N=64 (SD^a^)	Sex	
		Male, n=46 (SD)	Female, n=18 (SD)
Age, years	33.3 (7.7)	33.0 (7.9)	33.9 (7.1)
Height, cm	178.2 (11.0)	182.5 (9.0)	167.3 (7.9)
Body mass, kg	103.7 (24.8)	112.5 (21.7)	80.1 (16.7)
Resistance training experience, years	12.3 (7.9)	14.2 (7.8)	7.6 (6.3)
Strongman training experience, years	4.8 (3.6)	5.5 (3.4)	3.1 (3.4)
Strongman competition experience, years	4.0 (3.1)	4.4 (3.0)	2.9 (3.1)

^a^SD: standard deviation

**Table 2:** Test-retest reliability of continuous data for demographics and training practices of all strongman athletes. Intraclass correlation coefficient *P*<.001 for all values.

Test-retest reliability	n	Number of response options	ICC	95% CI	Qualitative inference
How many years of general resistance training experience do you have?	62^a^	41	.94	.90 to .96	Excellent
How many years of strongman implement training experience do you have?	62^a^	36	.97	.96 to .98	Excellent
How many years have you been competing in the sport of strongman?	64	32	.98	.96 to .99	Excellent
On average, how many days per week do you train?	63^b^	7	.86	.77 to .92	Excellent
On average, how many cardiovascular conditioning training sessions (includes both aerobic and anaerobic conditioning) do you perform per week?	64	16	.88	.81 to .93	Excellent
On average, how many resistance training sessions (includes both strongman and traditional training sessions) do you perform per week?	64	16	.84	.74 to .90	Excellent
On average, how long are your training sessions (to the nearest 15 minutes)?	64	13	.87	.79 to .92	Excellent

^a^n=64; valid=62; excluded=2

^b^n=64; valid=63; excluded=1

**Table 3:** Test-retest reliability of continuous data for strongman athletes who said they taper for strongman competitions (n=53).

Test-retest reliability	n	Number of response options	ICC (P-value)	95% CI	Qualitative inference
How many days would you consider your usual “taper” to be before a strongman competition?	43^a^	40	.30^f^	-.31 to .62	Fair
How many weeks out from a strongman competition do you train with the highest volume? (ie, sum of sets x repetitions x load)	43^a^	22	.72 (<.001)	.49 to .85	Good
How many weeks out from a strongman competition do you normally train with the highest intensity? (ie, highest load/degree of effort)	44^b^	11	.56 (.004)	.19 to .76	Moderate
What would be your estimated drop in your average training volume (as a percentage) during your taper?	37^c^	11	.77 (<.001)	.56 to .88	Good
How many days before a strongman competition do you cease to train?	43^a^	17	.81 (<.001)	.65 to .90	Excellent
How many days out before an important strongman event do you usually perform your final training session (at any weight)?	41^d^	15	.84 (<.001)	.70 to .92	Excellent
How many days before an important strongman event do you usually perform your final heavy training session (>85% 1RM)?	42^e^	15	.64 (.001)	.33 to .81	Good

^a^n=53; valid=43; excluded=10

^b^n=53; valid=44; excluded=9

^c^n=53; valid=37; excluded=16

^d^n=53; valid=41; excluded=12

^e^n=53; valid=42; excluded=11

^f^Poor reliability (adjusted categorical data is presented in Table 4);

The test-retest reliability of continuous data for strongman athletes who said they taper (n=53) for strongman competitions is reported in Table 3. Significant correlations were observed for all measures except for the number of days athletes considered their usual taper to be before a strongman competition (ICC=.30). Due to the importance of this question for the wider study, an additional analysis was conducted in which days were categorized into ranges (ie, <7, 7-10, 11-14, >14 days). The results of this analysis are included in Table 4.

Reliability was excellent for the number of days before a strongman competition that athletes ceased to train (ICC=.81) and the number of days out from an important strongman event when the final training session (at any weight) occurred (ICC=.84). Reliability was good for the number of days out from an important strongman event when the final heavy training session (>85% 1RM) occurred (ICC=.64). Good reliability was also observed for athletes’ estimated drop in average training volume (as a percentage) during the taper (ICC=.77) and for the weeks out from a competition in which they trained with the highest volume (ie, sum of sets x repetitions x load; ICC=.72). Reliability was moderate for the number of weeks out from a strongman competition that athletes normally trained with the highest intensity (ie, highest load/degree of effort; ICC=.56).

The test-retest reliability of categorical data for demographics, training practices, and tapering practices of strongman athletes is reported in Table 4. Kappa was significant for the majority of measures except for how training frequency (κ=.26) and the percentage and type of resistance training performed changed in the taper (κ=.20). Reliability was almost perfect for the highest level of competition athletes had competed at (κ=.85). Substantial reliability was observed for athletes indicating that they were self-coached or if they had a coach (κ=.66), if they tapered for strongman competitions (κ=.67), and if they always tapered for strongman competitions (κ=.73). Reliability was moderate for what the athletes’ usual resistance training looked like per week (κ=.45) and for how their training intensity (κ=.56) and training duration (κ=.48) changed throughout the taper. Moderate reliability was also observed in the additional analysis for the number of days athletes considered their normal taper to be (κ=.43). Reliability was fair for the type of tapering athletes used (κ=.38) and what the athletes’ cardiovascular training looked like per week (κ=.37).

**Table 4:** Test-retest reliability of categorical data for demographics, training practices, and tapering practices of strongman athletes.

Test-retest reliability		n	Number of response options	Kappa (asymptotic standard error)	P-value	95% CI	Qualitative Inference
**Demographics and Training Practices**							
	What is the highest level of strongman competition you have competed at?	64	4	.85 (.06)	<.001	.73 to 96	Almost perfect
	Are you self-coached or do you have a coach?	64	3	.66 (.07)	<.001	.53 to .80	Substantial
	On average, what does your usual resistance training look like per week?	63^a^	8	.45 (.08)	<.001	.30 to .60	Moderate
	On average, what does your cardiovascular training look like per week?	63^a^	9	.37 (.07)	<.001	.23 to .51	Fair
**Tapering and Tapering Practices**							
	Do you or have you ever used tapering when preparing for a strongman competition?	64	2	.67 (.12)	<.001	.43 to .92	Substantial
	How many days would you consider your usual “taper” to be before a strongman competition?	44^b^	4	.43^c^(.10)	<.001	.23 to .64	Moderate
	Which type of tapering do you use?	44^b^	4	.38 (.11)	<.001	.16 to .60	Fair
	Do you always use a taper before strongman competitions?	44^b^	2	.73 (.18)	<.001	.37 to 1.0	Substantial
	How does your training intensity change during your taper?	44^b^	3	.56 (.12)	<.001	.33 to .79	Moderate
	How does your training frequency change during your taper?	44^b^	3	.26 (.14)	.07	-.01 to .52	Fair
	How does your training duration (ie, time per training session) change during your taper?	44^b^	3	.48 (.13)	.001	.22 to .73	Moderate
	Does the percentage and type of resistance training you do (eg, percent traditional type training and percent strongman implement training) change in your taper?	44^b^	2	.20 (.15)	.19	-.09 to .49	Slight

^a^n=64; valid=63; excluded=1

^b^n=64; valid=44; excluded=20

^c^Kappa derived from the categorization of taper days (ie, <7, 7-10, 11-14, >14 days)

The test-retest reliability of continuous data relating to strongman events and traditional exercises is reported in Table 5. Significant correlations were observed for all measures except for the days before competition the Farmer’s Walk was performed, which showed poor reliability (ICC=.27).

Reliability was excellent for the loads used in the Yoke Walk (ICC=.91), Farmer’s Walk (ICC=.81), stone lifts/work (ICC=.81), and bench press (ICC=.93), and for the days before competition that the deadlift was performed (ICC=.87). Good reliability was observed for the loads used in the log lift/press (ICC=.74), deadlift (ICC=.73), squat (ICC=.69), and overhead presses (ICC=.71), and for the days before competition that the log lift/press (ICC=.62), Yoke Walk (ICC=.74), stone lifts/work (ICC=.75), squat (ICC=.73), overhead presses (ICC=.71), and bench press (ICC=.75) were performed.

**Table 5:** Test-retest reliability of continuous data relating to strongman events and traditional exercises. ICC analysis conducted when responses were n>11.

Test-retest reliability				n		ICC (P-value)		95% CI		Qualitative inference
**Could you please choose FIVE of your core strongman exercises and the corresponding days out from competition you would last perform the exercise and what loads you would use?**										
	**Log lift/press**									
		Days when last performed before competition	37		.62 (.003)		.25 to .81		Good	
		What loads were used	35		.74 (<.001)		.49 to .87		Good	
	**Yoke Walk**									
		Days when last performed before competition	32		.74 (<.001)		.47 to .87		Good	
		What loads were used	32		.91 (<.001)		.81 to .95		Excellent	
	**Farmer’s Walk**									
		Days when last performed before competition	28		.27 (<.001)		-.52 to .66		Poor	
		What loads were used	27		.80 (<.001)		.57 to .91		Excellent	
	**Stone lifts/work**									
		Days when last performed before competition	16		.75 (.01)		.28 to .91		Good	
		What loads were used	24		.81 (<.001)		.57 to .92		Excellent	
**Could you please choose FIVE of your core traditional exercises and the corresponding days out from competition you would last perform the exercise and what loads you would use?**										
	**Deadlift**									
		Days when last performed before competition	32		.87 (<.001)		.74 to .94		Excellent	
		What loads were used	31		.73 (<.001)		.43 to .87		Good	
	**Squat**									
		Days when last performed before competition	28		.73 (.001)		.42 to .87		Good	
		What loads were used	27		.69 (.003)		.31 to .86		Good	
	**Overhead presses**									
		Days when last performed before competition	15		.71 (.01)		.19 to .90		Good	
		What loads were used	14		.71 (.02)		.64 to .91		Good	
	**Bench press**									
		Days when last performed before competition	11		.75 (.03)		-.11 to .93		Good	
		What loads were used	11		.93 (<.001)		.72 to .98		Excellent	

## Discussion

This study examined the test-retest reliability of *The Tapering Practices of Strongman Athletes Survey* designed to determine how strongman athletes taper for strongman competitions. The results supported our initial hypothesis and indicated that the self-reported questionnaire, delivered using Internet commercial software, provided stable and reliable answers for the majority of measures. The sample of 64 athletes who participated in this study represents 14.1% of the 454 strongman athletes who participated in the wider *Tapering Practices of Strongman Athletes Survey* study (publication under review). Our sample size of 64 athletes is similar to (or higher than) other recent test-retest reliability studies recalling physical activity behaviors among specific populations [14,31,32].

Significantly high test-retest reliability results were observed for data relating to strongman demographics and training practices (ICCs=.84-.98). Researchers have found that items that assess habits have higher reliability scores than items assessing attitudes and awareness [33]. It is quite likely that because strongman training practices are repetitive behaviors, they may be more clearly remembered by strongman athletes.

Of the categorical data, only two items (training frequency and the percentage and type of resistance training performed changed in the taper) did not show significant agreement. The remaining items showed significance and demonstrated acceptable agreement. It is important to note that values for kappa rarely exceed .75 due to the adjustment for chance agreement [34]. Therefore, the categorical results relating to strongman training practices and tapering practices tended to exhibit favorable kappa values overall.

Only two items (days before competition the Farmer’s Walk is performed, and the number of days strongman athletes considered their usual taper to be) did not show significant reliability. The remaining items showed significance and exhibited moderate to excellent reliability values overall (ICCs=.56-.98). Another study utilizing an online survey reported that the Farmer’s Walk is the most commonly used exercise among strongman athletes (n=167) [1]. As such, it may be more difficult for athletes to recall exactly when the exercise was last used during the taper. Furthermore, every strongman competition is somewhat unique, which may affect the taper employed (ie, length of taper and training volume), thus making the recall of some taper activity more difficult. The four items in the current study (training frequency, percentage and type of resistance training performed, number days before competition the Farmer’s Walk is performed, and the length of taper) that did not show significant reliability related to specific questions on the taper. Researchers have suggested that recalling behaviors over limited time periods requires a more complex cognitive process than recalling behaviors over longer periods [35].

Due to the importance of quantifying the strongman athletes’ mean taper length and the poor reliability associated with the taper length described as a continuous variable in the current study, we conducted an additional analysis in which the data (in days) were categorized (ie, <7, 7-10, 11-14, >14 days). Significant moderate reliability (κ=.43; *P*<.001) was then observed for the number of days athletes considered their normal taper to be. These additional categorical analyses will be used in the wider study, as this is an effective approach for presenting important data rather than omitting the data due to poor reliability.

There were a number of limitations to the current study. The survey was open for 8 weeks and the participants exhibited some variation in the time between test and retest (mean 27.5 days, SD 14.1). Such an approach was warranted in this study, as many athletes were actively involved in competition and were competing overseas or in different states. If the exact time between test and retest was more stringent, a substantial loss of participants would likely have been observed. Leppink and Pérez-Fuster [36] have suggested that the length of the test-retest interval should be long enough that memory or practice effects can fade, and at the same time not too short for historical changes to occur on part of the respondent. The moderate and fair scores associated with what the strongman athletes’ usual resistance and usual cardiovascular training looked like per week (κ=.45 and κ=.37, respectively) may have been influenced by training regime changes over time. Another limitation of this study was insufficient power to allow us to explore differences between different subgroups of the sample. It would have been interesting to determine if differences in reliability measures existed between sex and competitive level.

In conclusion, *The Tapering Practices of Strongman Athletes* questionnaire is a low-cost instrument that is straight-forward to administer and provides stable and reliable answers. The questionnaire could easily be modified to fit the needs of other competitive weight lifting sports (ie, weightlifting, powerlifting, CrossFit, and Highland Games) and presents an effective online tool for assessing tapering practices leading up to competition. Further research could investigate how strongman athletes prepare themselves for strongman events on competition days and investigate strategies used for optimal arousal.

## Appendix

Multimedia Appendix 1 Questionnaire items tested for reliability.

## Abbreviations

1RM: one repetition maximum
ICC: intraclass correlation coefficient
SD: standard deviation
