# Supplementary material for: Tapering Practices of Strongman Athletes: Test-Retest Reliability Study
Source: JMIR Res Protoc. 2017 Oct 31;6(10):e211. doi: 10.2196/resprot.8522 (PMC5686420; doi:10.2196/resprot.8522)
Supplement: Multimedia Appendix 1 [file resprot_v6i10e211_app1.pdf]

Multimedia Appendix 1. Questionnaire items tested for reliability.

**Questionnaire Item**	**Possible responses**
Demographics and Training Practices	
How many years of general resistance training experience do you have?	1/2/3/4/5/6/7/8/9/10/11/12/13/14/15/16/17/18/19/20/21/22/23/24/25/26/27/28/29/30/31/32/33/34/35/36/37/38/39/40/>40
How many years of strongman implement training experience do you have?	1/2/3/4/5/6/7/8/9/10/11/12/13/14/15/16/17/18/19/20/21/22/23/24/25/26/27/28/29/30/31/32/33/34/35/>35
How many years have you been competing in the sport of strongman?	Never competed/<1/1/2/3/4/5/6/7/8/9/10/11/12/13/14/15/16/17/18/19/20/21/22/23/24/25/26/27/28/29/30
What is the highest level of strongman competition you have competed at?	Local amateur/regional amateur/national amateur/professional
Are you self-coached or do you have a coach?	Self-coached/Have a coach/Other - Please specify
On average, how many days per week do you train?	1/2/3/4/5/6/7
On average, how many cardiovascular conditioning training sessions (includes both aerobic and anaerobic conditioning) do you perform per week?	0/1/2/3/4/5/6/7/8/9/10/11/12/13/14/>14
On average, how many resistance training sessions (includes both strongman and traditional training sessions) do you perform per week?	1/2/3/4/5/6/7/8/9/10/11/12/13/14/15/>15
On average, how long are your training sessions (to the nearest 15 minutes)?	15/30/45//60/75/90/105/120/135/150/165/180/>180
On average, what does your usual resistance training look like per week?	Mostly traditional with 1 strongman session/Mostly traditional with 2 strongman sessions/Mostly traditional with 3 strongman sessions/Mostly combined strongman and traditional training/Mostly strongman with 3 traditional sessions/Mostly strongman with 2 traditional sessions/Mostly strongman with 1 traditional session/Other - Please specify:
On average, what does your cardiovascular training look like	I do not perform cardiovascular training/Mostly aerobic

per week?	with 1 anaerobic session/<br><br>Mostly aerobic with 2 anaerobic sessions/Mostly aerobic with 3 anaerobic sessions/<br><br>Mostly combined aerobic and anaerobic in each session/Mostly anaerobic with 3 aerobic sessions/Mostly anaerobic with 2 aerobic sessions/Mostly anaerobic with 1 aerobic session/Other - Please specify
Tapering Practices	
Do you or have you ever used tapering when preparing for a strongman competition?	Yes/no
How many days would you consider your usual ‘taper’ to be before a strongman competition?	<3/3/4/5/6/7/8/9/10/11/12/13/14/15/16/17/18/19/20/21/22/23/24/25/26/27/28/29/30/31/32/33/34/35/36/37/38/39/40/>40
Which type of tapering do you use?	The step taper/the linear taper/the exponential taper with a slow or fast decay/other
Do you always use a taper before strongman competitions?	Yes/no
How many weeks out from a strongman competition do you train with the highest volume? (ie, sum of sets x reps x load)	1/2/3/4/5/6/7/8/9/10/11/12/13/14/15/16/17/18/19/20/21/>21
How many weeks out from a strongman competition do you normally train with the highest intensity? (ie, highest load/degree of effort)	1/2/3/4/5/6/7/8/9/10/>10
What would be your estimated drop in your average training volume (as a percentage) during your taper?	10/20/30/40/50/60/70/80/90/>90/no change in training volume
How does your training intensity change during your taper?	Increases/stays the same/decreases
How does your training frequency change during your taper?	Increases/stays the same/decreases
How does your training duration (ie, time per training session) change during your taper?	Increases/stays the same/decreases
How many days before a strongman competition do you cease to train?	<1/1/2/3/4/5/6/7/8/9/10/11/12/13/14/15/>15

How many days out before an important strongman event do you usually perform your final training session (at any weight)?	1/2/3/4/5/6/7/8/9/10/11/12/13/14/>14
How many days before an important strongman event do you usually perform your final heavy training session (>85% 1RM)?	1/2/3/4/5/6/7/8/9/10/11/12/13/14/>14
Does the percentage and type of resistance training you do (eg, % traditional type training and % strongman implement training) change in your taper?	Yes/no
Could you please choose FIVE of these strongman exercises and the corresponding days out from competition you would last perform the exercise and what loads you would use?	Exercise: Farmers walk/log lift-press/sled-truck pull/yoke walk/stone lifts-work/axle lift-press/tyre flip/other<br><br>Days out from competition: 1/2/3/4/5/6/7/8/9/10/11/12/13/14/15/16/17/18/19/20/21/>21<br><br>Load (as a %of 1RM) <40/40/45/50/55/60/65/70/75/80/85/90/95/100
Could you please choose FIVE of your core traditional exercises and the corresponding days out from competition you would last perform the exercise and what loads you would use?	Exercise: Bench press/squat/deadlift/rows/Olympic lifts/overhead presses/assistance exercises<br><br>Days out from competition: 1/2/3/4/5/6/7/8/9/10/11/12/13/14/15/16/17/18/19/20/21/>21<br><br>Load (as a %of 1RM) <40/40/45/50/55/60/65/70/75/80/85/90/95/100
